# Magnitude of second-trimester-induced abortion and associated factors among women who received abortion service at public hospitals of Arba Minch and Wolayita Sodo towns, southern Ethiopia: A cross-sectional study

**DOI:** 10.3389/fgwh.2022.969310

**Published:** 2022-10-14

**Authors:** Mesfin Abebe, Abera Mersha, Nega Degefa, Wondwosen Molla, Aregahegn Wudneh

**Affiliations:** ^1^Department of Midwifery, College of Medicine and Health Sciences, Dilla University, Dilla, Ethiopia; ^2^School of Nursing, College of Medicine and Health Sciences, Arba Minch University, Arba Minch, Ethiopia

**Keywords:** second-trimester-induced abortions, associated factors, Ethiopia, magnitude, abortion service

## Abstract

**Introduction:**

Second-trimester abortion accounts for 10–15% of all induced abortions, with varying rates across countries, and is responsible for two-thirds of major abortion complications. It is also associated with higher medical costs, morbidity, and mortality rates than first-trimester abortion. Even though it is a significant burden, there is a lack of adequate information about second-trimester-induced abortion, especially in the study area. As a result, the primary purpose of this study is to fill this research gap and assess the magnitude and associated factors of second-trimester-induced abortion in the public hospitals of Arba Minch and Wolayita Sodo towns, southern Ethiopia.

**Methods:**

A facility-based cross-sectional study was conducted. Systematic sampling was used to select 353 study participants. Data were collected through face-to-face interviews using a structured questionnaire and record review by using Kobo collect version 3.1. Analysis was done by STATA 14. Logistic regression was used to identify associated factors of the second-trimester-induced abortion.

**Results:**

The magnitude of second-trimester-induced abortion in the study setting was 23% (95%CI: 18.5%, 27.4%). The factors associated with second-trimester-induced abortion among women received abortion care services were respondent's age 25–29 and 30–34 years old (AOR = 0.38, 95%CI:0.15, 0.96 and (AOR = 0.31, 95%CI:0.10, 0.97, respectively), planned pregnancy (AOR = 0.22, 95%CI:0.11, 0.44), and delay confirming pregnancy (AOR = 2.21, 95%CI:1.15, 4.23).

**Conclusion:**

This study showed that more than one-fifth of women who presented for abortion care services had second-trimester-induced abortions. Health institution organizations working on maternal health at various levels should provide counseling to women to help them early confirm their pregnancy and make decisions about whether or not to continue it as early as possible.

## Introduction

Second-trimester abortion is defined as the termination of pregnancy between the 13 and 28 weeks of gestational age; it is divided into two periods: an early period between 13 and 20 weeks, and a late period between 20 and 28 weeks (1, 2). Second-trimester abortion is an important component of comprehensive women's health care, and women tend to terminate later in pregnancy for a variety of medical and social reasons (3). Globally, from 2015 to 2019, approximately 73.3 million induced abortions were performed annually, with 45% of these abortions being performed unsafely. Almost half of these unsafe abortions occurred in developing countries, including Ethiopia (4). Developing countries accounted for more than 98% of all unsafe abortions (5). The majority of induced abortions occur in the first trimester, but second-trimester abortion accounts for 10–15% of all induced abortions, with varying rates across countries, and is responsible for two-thirds of major abortion complications (2, 6).

Abortion-related maternal deaths account for 13% of all maternal deaths worldwide. The majority of them are caused by unsafe abortions, with a large number of them occurring in the second trimester (7). More than 77% of induced abortions are terminated in unsafe conditions in sub-Saharan Africa and account for 50% of maternal deaths, and the abortion rate nearly doubled from 4.3 million to 8.0 million between 1995–1999 and 2015–2019 (8, 9). Second-trimester abortions have higher medical costs, morbidity, and mortality rates than first-trimester abortions (6).

Ethiopia is one of the low-income countries in sub-Saharan Africa with the highest maternal morbidity and mortality rates. The maternal mortality rate in Ethiopia was 412 maternal deaths per 100,000 live births, according to the 2016 Ethiopia Demographic and Health Survey (2016 EDHS) (10). If adequate abortion care is obtained promptly, the severity of complications arising from abortion may be minimized. Delays in providing care had long been recognized as the leading cause of maternal deaths (11). In Ethiopia, around 20–40% of women seeking abortion services during the second trimester are admitted after complications of unsafe abortion (12). Abortion-related mortality accounted for more than 30% of maternal deaths, with second-trimester abortion accounting for 11% of all maternal deaths (7). The second-trimester abortion care service is very minimal and only 9–10% of providers from all facilities can offer this service (12, 13). However, in some countries, including Ethiopia, the majority of abortion-related deaths were caused by unsafe second-trimester abortions (14). According to institution-based cross-sectional studies in Ethiopia's Amhara region, Jimma town, and Debre Markos, the magnitude of second-trimester-induced abortion was 19, 13.7, and 29.6%, respectively (2, 15, 16).

Some pieces of evidence have shown that young age, delayed pregnancy diagnosis, delayed decision, low educational status, irregular menstrual cycle, lack of information about where to get abortion care service, being unmarried, women employed in the private sector, failure of contraception difficulty arranging transportation, fear of stigma, and fear of abortion were contributing factors of second-trimester-induced abortion (2, 6, 16–18). Ethiopia made some strides in 2005 by revising the abortion law, which previously only allowed procedures to save a woman's life, and making safe abortion available to many women. Following that, abortion is legal whether the pregnancy is the result of rape or incest, the continuation of the pregnancy risks the mother's or child's health, the fetal disability is severe or incurable, and the woman is in a minority that is physically and mentally unprepared for childbirth (19).

Several efforts have been made to improve abortion-related services by the expansion of abortion services to primary healthcare units, and the development and dissemination of national guidelines for providing legal and safe abortion services (20). In 2014, the guideline was revised to update the gestational age limits for medication abortion and to make second-trimester abortion services more available (21). These efforts have resulted that abortion in health facilities increased to 53% in 2014 from 27% in 2008, and induced abortion and post-abortion care provided by mid-level providers increased to 53% in 2014 from 27% in 2008 (22).

Even with numerous initiatives and attempts to increase access to safe abortion facilities, nearly six out of ten abortions in Ethiopia are still conducted in a risky manner, and second-trimester abortion is an emerging issue (23). Second-trimester abortion has a greater risk of morbidity and mortality than first-trimester abortion and causes perforation, rupture, inflammation, and hemorrhage of the uterus. A further enhancement is still needed; so assessing its magnitude is important for tracking progress toward sustainable development goals. Despite the fact that it is a significant burden, there is a lack of adequate information about second-trimester-induced abortion, especially in the study area. As a result, this study aims to determine the magnitude of induced second-trimester abortion and associated factors in public hospitals of Arba Minch, and Sodo town, southern Ethiopia.

## Materials and methods

### Study setting and period

In this study, women who received abortion services in public hospitals of Arba Minch and Wolayita Sodo towns, southern Ethiopia were involved from 25 April to 25 July, 2021. Arba Minch and Wolayita Sodo towns are situated 505 and 330 km south of Addis Ababa and are the administrative center of the Gamo zone and Wolayita zone, respectively. According to estimates from the 2007 central statistical agency survey, Arba Minch general hospital serves over 1.6 million people in Arba Minch town. Whereas Wolayita Sodo University teaching and referral hospital gives a wide range of medical services for around 2 million people in and outpatients of all age groups (24). According to the reports of zonal health offices, Arba Minch and Wolayita Sodo towns have three public hospitals (one general hospital, one teaching and referral hospital, and one primary hospital) and five health centers.

### Study design

An institutional-based cross-sectional study was conducted to meet study objectives.

### Population

#### Source population

All women of childbearing age who visited the Arba Minch general hospital, and Wolayita Sodo University teaching referral hospital for abortion care services.

#### Study population

All women of childbearing age who visited the Arba Minch general hospital, and Wolayita Sodo University teaching referral hospital for abortion care services during the data collection period.

#### Sample population

All selected women of childbearing age who received abortion care in Arba Minch general hospital, and Wolayita Sodo University teaching referral hospital during the data collection period.

### Eligibility criteria

The inclusion criteria were all women who received abortion care in selected health facilities and women who presented with complications due to induced abortion conducted outside health institutions will be included. Whereas the exclusion criteria were women who request abortion after the gestational age of 28 weeks, and those who are unable to communicate or seriously ill during the data collection period.

### Sample size determination

Open Epi info version 3 software was used to estimate the sample sizes. For the first objective, a single population proportion was used by considering the following assumption: the proportion of the second-trimester-induced abortion (*P* = 0.296) from the study conducted in Ethiopia (16), standard normal distribution at 95% of confidence level, and margin of error of 5%. Based on this assumption, the calculated sample size was 321. Therefore, the sample used for this study was 353 by adding a non-response rate of 10% to the larger sample size.

### Sampling procedure

**Figure 1:**
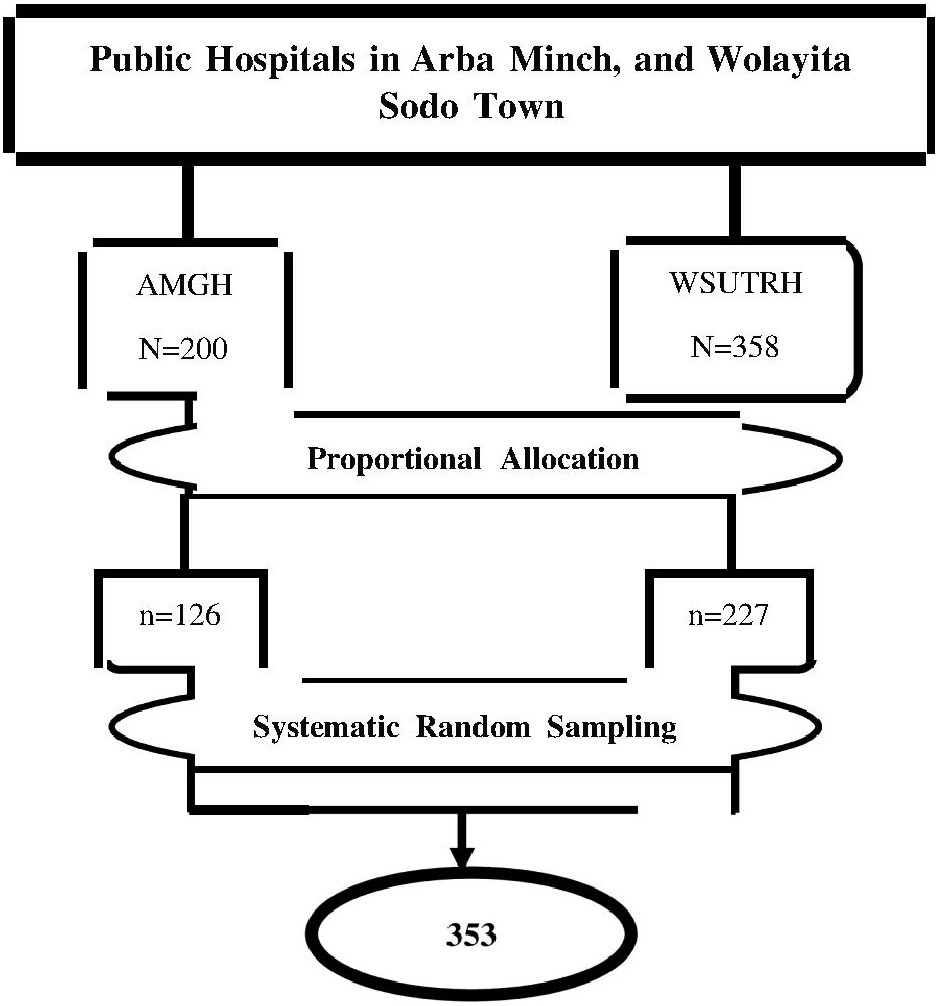
Schematic presentation of sampling procedures to assess the magnitude and associated factors of second trimester induced abortion women who received abortion services at public hospitals in Arba Minch, and Wolayita Sodo town, southern Ethiopia, 2021. Where: AMGH, Arba Minch General Hospital, WSUTRH, Wolayita Soda University Teaching Referral Hospital.

Recently, there are three public hospitals in Arba Minch and Wolayita Sodo towns. From them, Arba Minch general hospital and Wolayita Sodo University teaching and referral hospital were included. By reviewing the previous year's 2-month report at the time of the data collection period, the sample for each hospital was arranged based on their patient flow. After the proportional allocation of samples for each hospital, a systematic sampling technique was used to select study subjects, and participants were interviewed after receiving all necessary abortion care (Figure 1).

### Data collection tools and procedures

Data were collected using a standardized and pretested interviewer-administered questionnaire, which is adapted from previous related studies (2, 6, 15, 16). The questionnaires contain four sections: Sociodemographic characteristics, reproductive health-related, health care utilization-related factors, and intrapersonal and interpersonal factors. Three BSc holder midwives and two MSc holders were recruited for data collectors and supervisory activities, respectively.

An exit face-to-face interview and record review were used to collect data. Kobo collects the version 3.1 mobile tool used to collect the data. The training was given to data collectors and supervisors on data collection tools, interview techniques, the confidentiality of information, objective, and the relevance of the study by the principal investigator.

### Study variables

The second-trimester-induced abortion was the dependent variable for this study. The independent variables were socio-demographic and economic characteristics (age, residence, marital status, educational and occupational status, and average monthly income), reproductive health-related factors, health care utilization, and intrapersonal and interpersonal-related factors.

### Operational definitions

**Induced abortion:** Post-abortion patients who reported intentionally terminating their pregnancy, either on their own or with the help of another (25). **Second-trimester-induced abortion:** Women having induced abortion at or after the gestation of 13 completed weeks up to 28 weeks of gestation (6). **Second-trimester pregnancy (STM):** Pregnancy with a gestational age of 13–27 weeks as determined by the attending healthcare professionals (12). **First-trimester pregnancy (FTM):** Pregnancy with a gestational age of less than 13 weeks as determined by the attending healthcare professionals (12).

### Data quality management

The questionnaire was initially prepared in English, then translated to the local language, and then translate back to English to check the consistency. The training was given to supervisors and data collectors for 2 days. The Kobo collect mobile tool that was very important to control the quality of data. Three weeks before the actual data collection pretest was conducted in randomly selected 5% of the calculated final sample size at the Sawla general hospital to ensure the consistency of the tool by the principal investigators. Before uploading the data to the Kobo collect cloud server, the principal investigator and supervisors double-checked it for completeness. All the data were checked for completeness and consistency during data management, storage, and analysis.

### Data processing and analysis

The collected data were downloaded from the Kobo server and exported to the SPPS version 25 for editing, cleaning, coding, and ensuring completeness and accuracy then exported to STATA 14 for analysis. A descriptive analysis was done to describe the pertinent characteristics of our study participants. After that, simple frequencies, and summary measures be used to present the data. Both bivariate and multivariable analyzes were used to assess the association between each independent variable and the outcome variable by using binary logistic regression. Variables with a 95% confidence interval and *P* < 0.25 during the bivariate analysis were included in the multivariable logistic regression analysis to control all potential confounding variables. In addition, even if the above parameters were not met, variables that were significant in previous studies and from a contextual viewpoint were included in the final model. Hosmer and Lemeshow's goodness of fitness test was used to check model fitness (*P* > 0.05). Adjusted odds ratios with a 95% confidence interval were calculated, and a *P* < 0.05 was considered statistically significant. Finally, data were presented using tables, graphs, and texts.

## Results

### Socio-demographic and economic characteristics of study participants

Out of 353 study participants, who received abortion care services, 352 women completed the face-to-face interview administered questionnaire with a response rate of 99.7%. The mean and standard deviation of respondents' age was 25.75 ± 5.1 years. Of the respondents, married women constitute 230 (65.34%) and those who live in the urban area constitute 217 (61.65%). In their ethnicity, Wolayita comprises 180 (51.14%).

The predominant religion was Protestant 173 (49.15%). Among educational and occupational status, 142 (40.34%) had secondary and 103 (29.26%) were housewives, respectively. Regarding marital status, 230 (65.34%) respondents were married. The median monthly income was 2,500 (IQR: 1000, 4000) Ethiopia Birr (Table 1).

**Table 1:** Socio-demographic and economic characteristics of women who received abortion care services in public hospitals of Arba Minch and Wolayita Sodo town, southern Ethiopia, 2021.

**Variables**	**Frequency**	**Percentage (%)**
**Respondents age (in year)**
15–19	44	12.50
20–24	75	21.31
25–29	151	42.90
30–34	52	14.77
≥35	30	8.52
**Religion**
Protestant	173	49.15
Orthodox	143	40.63
Catholic	21	5.97
Muslim	15	4.26
**Ethnicity**
Wolayita	180	51.14
Gamo	110	31.25
Amhara	23	6.53
Gofa	23	6.53
Other*	16	4.54
**Marital status**
Single	92	26.14
Married	230	65.34
Other^**±**^	30	8.52
**Educational status**
No formal education	62	17.61
Primary education	81	23.01
Secondary education	142	40.34
Diploma and above	67	19.03
**Occupational status**
Government employee	75	21.31
Merchant	78	22.16
Student	50	14.20
House wife	103	29.26
Other^®^	46	13.07
**Monthly income**
<2,000 ETB	159	45.17
2,001–4,000 ETB	110	31.25
≥4,001 ETB	83	23.58

*Konso, Oromo, and Tigre, ^**±**^Divorced, separated and widowed,^®^unemployed, and daily labor.

### Magnitude of the second-trimester-induced abortion

In this study, the magnitude of second-trimester-induced abortion in Arba Minch general hospital and Wolayita Sodo University teaching and referral hospital was 81 (23%) (95%CI: 18.5%, 27.4%).

### Reasons for delay in seeking abortion care services

Out of 89 induced abortions, 81 respondents were delayed for various reasons, including 38 (46.91%) being delayed due to unrecognized pregnancy early, 19 (23.46%) being confused to terminate or continue by peer pressure, 10 (12.35%) taking a long time discussing abortion with family/partner, 8 (9.88%) not expecting the relationship with their husband/partner changed, and 6 (7.41%) were taking a long time because they are afraid to tell their families.

### Reproductive characteristics

Out of 352 respondents, 216 (61.36%) had a regular menstrual cycle before the current abortion. Nearly 26% of the respondents were pregnant for the first time. Eighty-nine (25.28%) of the respondents did not have alive children. Eighty (22.73%) women had a previous history of abortion (Table 2).

**Table 2:** Reproductive characteristics of women who received abortion care services in public hospitals of Arba Minch and Wolayita Sodo towns, southern Ethiopia, 2021.

**Variables**	**Frequency**	**Percentage (%)**
**Nature of menses**
Regular	216	61.36
Irregular	136	38.64
**Number of pregnancy**
1	91	25.85
2	123	34.94
3	78	22.16
4	36	10.23
≥5	24	6.82
**Number of delivery**
Nullipara	86	24.43
1	124	35.23
2	85	24.15
3	35	9.94
≥4	22	6.25
**Number of alive children**
0	89	25.28
1	128	36.36
2	78	22.16
3	37	10.51
≥4	20	5.68
**Contraceptive used history**
Yes	279	79.26
No	73	20.74
**Types of contraceptive used**
Emergency pills	15	5.38
Oral contraceptives pills	41	14.70
Injectable	111	39.78
Implants	94	33.69
IUCD	9	3.23
Unspecified	9	3.23
**Pregnancy status**
Planned	248	70.45
Unplanned	104	29.55
**Know last normal menstrual period (LNMP)**
Yes	183	51.99
No	169	48.01
**Know sign and symptoms of pregnancy**
Yes	125	35.51
No	227	64.49
**Delay to confirm pregnancy from LNMP**
Yes	97	27.56
No	255	72.44

### Health care utilization related factors

About 123 (34.94%) of the respondents took a long time to get money for abortion care services. One hundred four (29.55%) of women had transportation problems to reach health institutions to get services (Table 3).

**Table 3:** Health care utilization of women who received abortion care services in public hospitals of Arba Minch and Wolayita Sodo town, southern Ethiopia, 2021.

**Variables**	**Frequency**	**Percentage (%)**
**Difficult to get abortion service provider**		
Yes	279	79.26
No	73	20.74
**Information about where abortion service is available**		
Yes	328	93.18
No	24	6.82
**Referral from other health institution**		
Yes	212	60.23
No	140	39.77

### Intrapersonal and interpersonal characteristics of study participants

Eighty (22.73 %) of respondents faced difficulty deciding whether or not to have an abortion. About 292 (82.95%) of respondents were not allowed abortion in their religion. Three hundred eleven (88.35%) of the respondents were afraid of having an abortion, while 101 (28.69%) of the participants faced opposition from family/friends to continue their pregnancy.

### Factors associated with second-trimester-induced abortion

The respondents' age, residence, educational status, nature of menses, previous history of abortion, pregnancy status, delay to confirm pregnancy, and opposition from husband/family were candidate variables for multivariable logistic regression. In multivariable logistic regression, respondents' age, pregnancy status, and delay confirming pregnancy were significantly associated with second-trimester-induced abortion. However, residence, educational status, nature of menses, previous history of abortion, and opposition from husband/family were not associated.

Those aged 25–29 and 30–34 years old were 62% and 69% less likely to have a second-trimester-induced abortion as compared with women whose age ranged from 15 to 19 years old (AOR = 0.38; 95%CI: 0.15, 0.96) and (AOR = 0.31; 95%CI:0.10, 0.97), respectively. The pregnancy status was planned 78% less likely to have induced second-trimester abortion as compared with an unplanned pregnancy (AOR = 0.22; 95%CI: 0.11, 0.44). The women who delay confirming their pregnancy were 2.21 times more likely to have the second-trimester-induced abortion than those who were not delayed confirming their pregnancy (AOR = 2.21; 95%CI: 1.15, 4.23) (Table 4).

**Table 4:** Bivariate and multivariable analysis of women who received abortion care services in public hospitals of Arba Minch and Wolayita Sodo towns, southern Ethiopia, 2021.

**Variables**	**Induced second trimester abortion**		**Crude OR**	**Adjusted OR**	***P*-value**
	**Yes**	**No**	**95%CI**		
**Respondents age (in year)**
15–19	20 (24.69%)	24 (8.86%)	1	1	
20–24	20 (24.69%)	55 (20.30%)	0.43 (0.19,0.95)	0.97 (0.38,2.51)	0.964
25–29	25 (30.86%)	126 (46.49%)	0.23 (0.11,0.49)	0.38 (0.15,0.96)*	**0.041**
30–34	8 (9.88%)	44 (16.23%)	0.21 (0.08,0.56)	0.31 (0.10,0.97)*	**0.045**
≥35	8 (9.88%)	22 (8.12%)	0.43 (0.15,1.19)	0.80 (0.24,2.62)	0.718
**Residence**
Urban	61 (75.31%)	156 (57.56%)	2.24 (1.28,3.93)*	1.69 (0.87,3.30)	0.11
Rural	20 (24.69%)	115 (42.44%)	1	1	
**Educational status**
No formal education	9 (11.11%)	53 (19.56%)	1	1	
Primary	20 (24.69%)	61 (22.51%)	1.93 (0.81,4.60)	1.08 (0.40,2.87)	0.87
Secondary	29 (35.80%)	113 (41.70%)	1.51 (0.66,3.41)	0.76 (0.29,1.96)	0.57
Diploma and above	23 (28.40%)	44 (16.23%)	3.07 (1.29,7.33)	2.27 (0.80,6.45)	0.12
**Nature of menses**
Regular	44 (54.32%)	172 (63.47%)	1	1	
Irregular	37 (45.68%)	99 (36.53%)	1.46 (0.88,2.41)	1.06 (0.59,1.90)	0.84
**Previous history of abortion**
Yes	23 (28.40%)	57 (21.03%)	1.48 (0.84,2.61)	0.77 (0.27,2.15)	0.62
No	58 (71.60%)	214 (78.97%)	1	1	
**Pregnancy status**
Planned	38 (46.91%)	210 (77.49%)	0.25 (0.15,0.43)	0.22 (0.11,0.44)*	**<0.001**
Unplanned	43 (53.09%)	61 (22.51%)	1	1	
**Delay to confirm pregnancy**
Yes	32 (39.51%)	65 (23.99%)	2.06 (1.22,3.50)	2.21 (1.15,4.23)*	**0.01**
No	49 (60.49%)	206 (76.01%)	1	1	
**Opposition from husband**
Yes	29 (35.80%)	72 (26.57%)	1.54 (0.90,2.61)	1.27 (0.50,3.24)	0.60
No	52 (64.20%)	199 (73.43%)	1	1	

*Significant at *P*-value <0.05. Bold to indicate variables that are statistically significant.

## Discussion

This facility-based cross-sectional study was conducted to determine the magnitude and associated factors of second-trimester-induced abortion in the public hospitals of Arba Minch and Wolayita Sodo towns. Accordingly, women's age, pregnancy status, and delay in confirming pregnancy were statistically significantly associated with second-trimester-induced abortion.

In this study, the magnitude of the second-trimester-induced abortion was 23% (95%CI: 18.5%, 27.4%). This finding was in line with studies done in the Amhara region, Ethiopia (19.2%) (2) and Harar, Ethiopia (18.2%) (26). The prevalence of induced second-trimester abortion in this study was lower compared with studies done in Debre Markos, Ethiopia (29.6%) (16), Kenya (39%) (27), and Chicago (32%) (28). The possible explanation for such observed difference might be a difference in sample size, study setting, and years of study.

However, the finding of this study is higher than the global reported prevalence of 10–15% and studies done in England and Wales (10%) (29), Texas (14.5%) (30), and Jimma Ethiopia (13.7%) (15). The possible explanation for this significant magnitude of second-trimester-induced abortion in the current study could be most non-governmental health institutions, private clinics, health centers, and primary hospitals are not providing abortion care services beyond 12 weeks of gestational age.

According to the current study, women between the ages of 25 and 29 were 62% less likely than those between the ages of 15 and 19 to have an induced abortion in the second trimester, and women between the ages of 30 and 34 were 69% less likely than those between 15 and 19 years old to have an induced abortion in the second trimester. This finding was in line with studies done in central Ethiopia (6), Kenya (27), and Netherland (31). As a result, adolescents are more likely to seek abortion services in the second trimester than adults. This may be explained by the lack of awareness among younger age about abortion-related health services, as well as the fear of seeking services during the early pregnancy period.

The odds of having a second-trimester-induced abortion were 78% less likely among women having wanted pregnancy compared with women having an unwanted pregnancy. This was congruent with a study done in Burkina Faso (32). The possible explanation could be women committing induced abortions most of the time from unwanted or unplanned pregnancies. Another possible explanation might be that abortion is legal in Ethiopia under certain preconditions, but still is stigmatized and not openly discussed in their families/communities.

The odds of second-trimester-induced abortion were 2.21 times more likely with women who delay in confirming/diagnosing their pregnancy compared with women who were not delayed in confirming pregnancy. This was in line with studies done in the Amhara region (2) and Central Ethiopia (6). The reason for this could be that women are unaware of the presence of pregnancy tests. Another possible explanation might be due to women waiting much time to get money and a lack of information about where services are available for pregnancy tests.

The main limitation of the study was that it could be subjected to recall bias. Because the study was cross-sectional, the causal association was under caution. This study may not be generalizable to the whole population. However, the current study investigated and provided useful input for a future multidimensional and multicenter study on the magnitude and determinants of the second-trimester-induced abortion.

## Conclusion

This study showed that more than one-fifth of women who presented for abortion care services at Arba Minch general hospital and Wolayita Sodo university referral and teaching hospital had second-trimester-induced abortion. Various delaying factors were preventing women from obtaining early abortion services. Women between the ages of 15 and 19, women who are unable to confirm their pregnancy early, and unplanned pregnancies were found to have abortions during the second-trimester period. As such, the investigators recommended that health institution organizations working on maternal health at various levels should provide counseling to women to help them early confirm their pregnancy and make decisions about whether or not to continue it as early as possible. Increasing accessibility of family planning options especially for those women who wanted no more children or unplanned pregnancies. Further qualitative research is required on the underlying factors that lead women to terminate their pregnancy in the second-trimester period.

## Data availability statement

All data included in this article are available upon request from the corresponding author via the email address.

## Ethics statement

The studies involving human participants were reviewed and approved by Institutional Research Ethics Review Board (IRB), College of Medicine and Health Sciences, Arba Minch University. Written informed consent to participate in this study was provided by the participants' legal guardian/next of kin.

## Author contributions

A designed the study, was involved in data collection, analysis, and interpretation of the result and drafted the article, and participated in preparing all versions of the manuscript. AM, ND, WM, and AW assisted in the design and the proposal development, monitored data collection, assisted during analysis, and revised subsequent drafts of the article. All authors have read and approved the final manuscript.

## Funding

Arba Minch University provided funds for the data collection and stationary materials of this research work with a project grant code of Acct. No. GOV-1000021480502. The website of the university is www.amu.edu.et. The funders had no role in study design, data collection, and analysis, decision to publish, or preparation of the manuscript.

## Conflict of interest

The authors declare that the research was conducted in the absence of any commercial or financial relationships that could be construed as a potential conflict of interest.

## Publisher's note

All claims expressed in this article are solely those of the authors and do not necessarily represent those of their affiliated organizations, or those of the publisher, the editors and the reviewers. Any product that may be evaluated in this article, or claim that may be made by its manufacturer, is not guaranteed or endorsed by the publisher.
